# Association of the Geriatric Nutritional Risk Index with the survival of patients with non-small-cell lung cancer after platinum-based chemotherapy

**DOI:** 10.1186/s12890-021-01782-2

**Published:** 2021-12-11

**Authors:** Masato Karayama, Yusuke Inoue, Hideki Yasui, Hironao Hozumi, Yuzo Suzuki, Kazuki Furuhashi, Tomoyuki Fujisawa, Noriyuki Enomoto, Yutaro Nakamura, Naoki Inui, Takafumi Suda

**Affiliations:** 1 Department of Chemotherapy, Hamamatsu University School of Medicine, 1-20-1 Handayama, Hamamatsu, 431-3192 Japan; 2 Second Division, Department of Internal Medicine, Hamamatsu University School of Medicine, 1-20-1 Handayama, Hamamatsu, 431-3192 Japan; 3 Department of Clinical Pharmacology and Therapeutics, Hamamatsu University School of Medicine, 1-20-1 Handayama, Hamamatsu, 431-3192 Japan

**Keywords:** Albumin, Cachexia, Hypoalbuminemia, Malnutrition, Nutrition

## Abstract

**Background:**

The nutritional status can potentially affect the efficacy of cancer therapy. The Geriatric Nutritional Risk Index (GNRI), a simple index for evaluating nutritional status calculated from body weight and serum albumin levels, has been reported to be associated with the prognosis of various diseases. However, the relationships between GNRI and the efficacy of platinum-based chemotherapy in patients with non-small-cell lung cancer (NSCLC) are unknown.

**Methods:**

The pretreatment levels of GNRI were retrospectively evaluated in 148 chemo-naïve patients with advanced NSCLC who received first-line platinum-based chemotherapy and scored as low or high.

**Results:**

Patients with a high GNRI had a significantly higher overall response rate (ORR; 44.5% [95% confidence interval {CI} = 35.6%–53.9%] vs. 15.8% [95% CI = 7.4%–30.4%, *p* = 0.002), longer median progression-free survival (PFS; 6.3 months [95% CI = 5.6–7.2 months] vs. 3.8 months [95% CI = 2.5–4.7 months], *p* < 0.001), and longer median overall survival (OS; 22.8 months [95% CI = 16.7–27.2 months] vs. 8.5 months [95% CI = 5.4–16.0 months], *p* < 0.001) than those with low GNRI. High GNRI was independently predictive of better ORR in multivariate logistic regression analysis and longer PFS and OS in multivariate Cox proportional hazard analyses. In 71 patients who received second-line non-platinum chemotherapy, patients with high GNRI exhibited significantly longer PFS and OS than those with low GNRI (both *p* < 0.001).

**Conclusions:**

GNRI was predictive of prolonged survival in patients with NSCLC who received first-line platinum-based chemotherapy and second-line non-platinum chemotherapy. Assessment of the nutritional status may be useful for predicting the efficacy of chemotherapy.

**Supplementary Information:**

The online version contains supplementary material available at 10.1186/s12890-021-01782-2.

## Introduction

Novel anti-cancer agents, such as oncogene-targeted drugs and immune checkpoint inhibitors (ICIs), have dramatically changed cancer therapy in recent decades. Meanwhile, the nutritional status is universally important for improving outcomes in patients with cancer. The associations between the nutritional status and prognosis are well established in a wide variety of non-cancer diseases, but they are particularly prominent in advanced cancer [1–5]. For example, cancer cachexia, characterized by the loss of body weight and lean body mass, leads to decreased quality of life, reduced chemotherapy tolerance, reduced physical function, and shortened survival [6–9]. Recently, anamorelin, a novel oral ghrelin receptor agonist, was revealed to improve cancer cachexia, and it has been approved for clinical use in patients with several cancers [10]. The nutritional status has been attracted renewed attention in cancer therapy.

The nutritional status does not merely represent a vague health status, but it is also associated with the mechanisms underlying tumor growth, anti-tumor activity of cancer therapy, or resistance to cancer therapy. For example, fat tissue-associated factors, such as leptin, fatty acids, and pro-inflammatory cytokines, contribute to cancer immunity [11]. In addition, serum albumin binds to chemotherapeutic agents and delivers them to tumor tissues [12–14]. The clinical benefits of cancer therapy are achieved through both effective anti-cancer therapy and preservation of the host status. Thus, the nutritional status can potentially predict the efficacy of cancer therapy.

The Geriatric Nutritional Risk Index (GNRI), a simple index for evaluating nutritional status calculated from body weight and serum albumin levels, has been reported to be useful for predicting the prognosis of various diseases, including infectious and chronic diseases [15–19]. In cancer therapy, the GNRI is reported to be associated with the clinical outcomes of surgery, chemotherapy, or chemoradiotherapy in a wide variety of cancers [20–22]. Furthermore, although GNRI was originally developed for elderly patients, it is also applicable for younger populations [23–25]. However, little is known about the association of the GNRI with the efficacy of chemotherapy in non-small-cell lung cancer (NSCLC). [26–29]. Body weight, the other component of the GNRI, is associated with cancer immunity in the tumor microenvironment via factors associated with fat tissue [11]. Thus, the GNRI might be predictive of the efficacy of chemotherapy. Platinum-based chemotherapy has been the standard treatment option for advanced NSCLC. Its efficacy and safety have a major impact on the subsequent second-line therapy and the overall survival of patients with NSCLC. The current study evaluated the pretreatment GNRI and its associations with the efficacy of first-line platinum-based chemotherapy and second-line non-platinum chemotherapy in patients with NSCLC.

## Patients and methods

### Study design

This was a retrospective observational study conducted in accordance with the ethical standards of the Declaration of Helsinki. Chemotherapy-naïve patients with pathologically confirmed advanced NSCLC who received first-line platinum-based chemotherapy at Hamamatsu University Hospital between January 1, 2000 and December 31, 2020 were included. Eligible patients were required to have stage IIIB without an indication for definitive radiotherapy, stage IV disease, or recurrent disease. Patients who received platinum-based chemotherapy as adjuvant treatment after surgery or in combination with radiotherapy (chemoradiotherapy), those who received combination therapy with platinum-based therapy and ICIs, those who received non-platinum therapy in the first-line setting, those with histories of previous chemotherapy including adjuvant chemotherapy, or those with missing pretreatment serum albumin and body weight data were excluded. Patient consent was waved because this was a retrospective study. The study protocol was approved by the Institutional Review Board of Hamamatsu University School of Medicine (No. 21-151).

### Data collection

Clinical data including age, sex, smoking status, height, weight, serum albumin levels before the administration of platinum-based chemotherapy, tumor histology, active driver mutations, clinical stage, Eastern Cooperative Oncology Group performance status (ECOG-PS), comorbidities, and treatment regimens were retrospectively evaluated via medical record reviews. Comorbidities were recorded according to the Charlson comorbidity index [30]. Response was assessed using Response Evaluation Criteria in Solid Tumors version 1.1. Progression-free survival (PFS) and overall survival (OS) were evaluated from the time of treatment initiation. The data cutoff date was August 31, 2021.

### Measurements of the Geriatric Nutritional Risk Index

The GNRI was calculated as follows:

$$\begin{aligned} {\text{GNRI}} & = \left[ {{\text{1}}.{\text{489}} \times {\text{serum albumin }}\left( {{\text{g}}/{\text{dL}}} \right)} \right] \\ & \quad + \left[ {{\text{41}}.{\text{7}} \times {\text{actual weight}}/{\text{ideal weight}}} \right]\;\left[ {{\text{15}}} \right]. \\ \end{aligned}$$

Ideal weight was calculated using body mass index as follows:

$${\text{Ideal weight}} = {22} \times \left[ {{\text{height }}\left( {\text{m}} \right)} \right]^{{2}} .$$

Originally, the GNRI was divided into four levels: < 82, ≥ 82 to < 92, ≥ 92 to < 98, and ≥ 98 [15]. However, in the current study, the GNRI was categorized as low (< 92) or high (≥ 92) because the overall response rate (ORR), PFS, and OS did not differ among the four GNRI categories (Additional file 1: Fig. S1).

### Statistical analyses

Fisher’s exact test and Wilcoxon’s rank sum test were used to compare categorical and continuous variables, respectively. Pearson’s correlation coefficient was used to evaluate the correlations between age and the GNRI. The Jonckheere–Terpstra trend test was used to evaluate the trend between the GNRI and ECOG-PS. Wilcoxon’s signed-rank sum test was used to compare the GNRI between first- and second-line therapy. PFS and OS were evaluated using the Kaplan–Meier method, and the log-rank test was used to compare survival curves. Logistic regression analysis was used to determine predictive factors for ORR, and Cox proportional hazard analysis was used to determine predictive factors for PFS and OS. The variables significant at *p* < 0.100 in univariate analyses were employed for multivariate analyses. *p* < 0.05 (two-sided) denoted significance. All values were analyzed using JMP v13.2.0 (SAS Institute Japan, Tokyo, Japan), excluding the Jonckheere–Terpstra test, which was performed using EZR (Saitama Medical Center, Jichi Medical University, Saitama, Japan), a graphical user interface for R (The R Foundation for Statistical Computing, Vienna, Austria).

## Results

### Patient characteristics

**Fig. 1:**
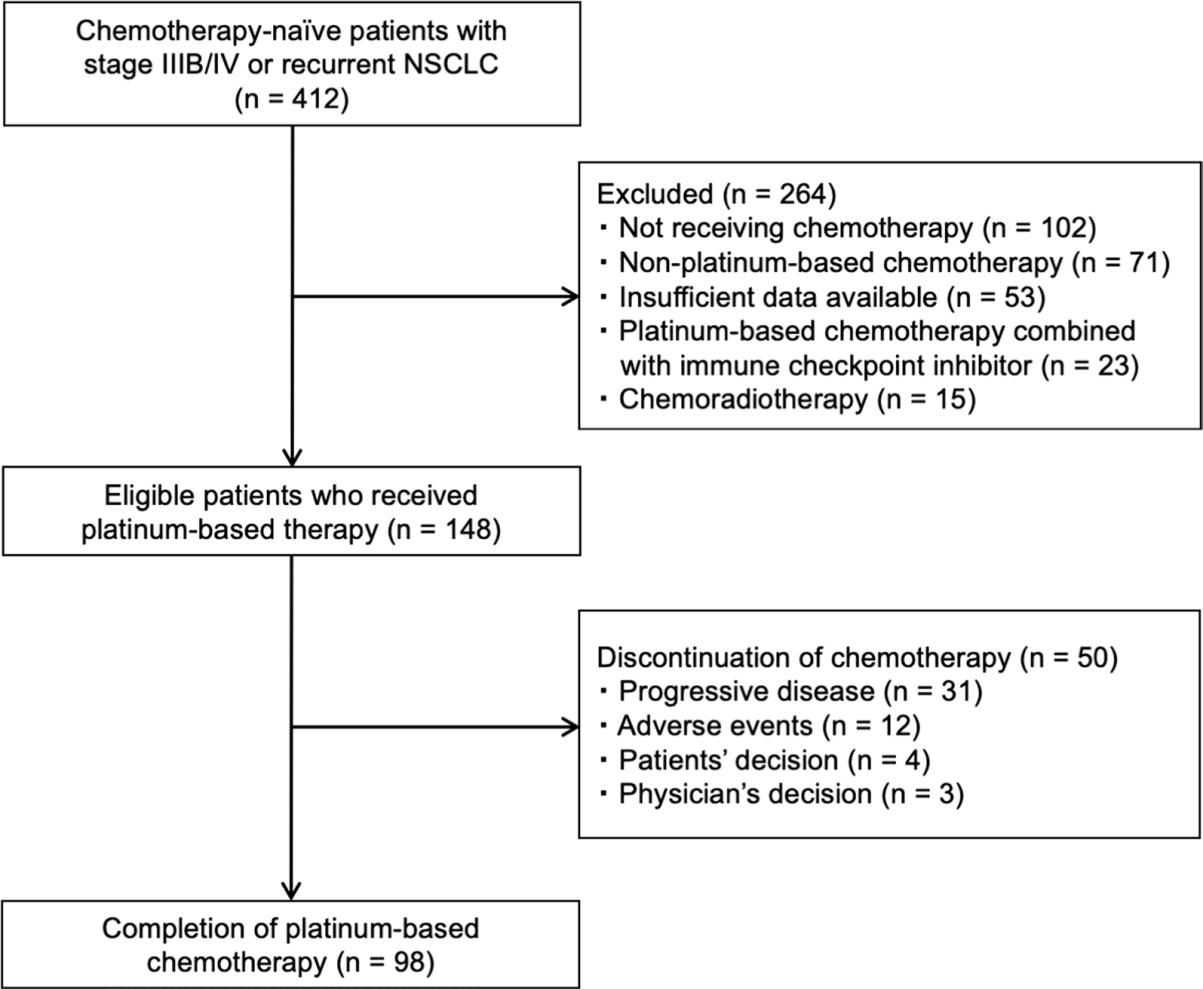
Diagram of study patients. *NSCLC* non-small cell lung cancer

In total, 412 patients were screened, and 264 were excluded because they did not receive chemotherapy (n = 102), they received non-platinum-based chemotherapy (n = 71), they had insufficient available data (n = 53), or they received platinum-based chemotherapy in combination with other treatments (n = 38). As the results, 148 patients were included in this study (Fig. 1). The characteristics of the patients are presented in Table 1. The cohort had a high proportion of men (73.6%), smoking history (75.0%), and good ECOG-PS of 0–1 (89.2%). Sixty-seven (45.2%) patients were ≥ 65 years old. Ninety-five (64.1%) patients had normal BMI (≥ 18.5 to < 25.0 kg/m^2^), and 26 (17.6%) and 27 patients (18.2%) were underweight and overweight, respectively. The median GNRI was 100.6 (range 56.6–124.6), and 19 (12.8%), 19 (12.8%), 22 (14.9%), and 88 (59.5%) patients had GNRIs of < 82, ≥ 82 to < 92, ≥ 92 to < 98, and ≥ 98, respectively. All patients received at least one cycle of platinum-based chemotherapy, and 119 (80.4%) and 29 patients (19.6%) received carboplatin and cisplatin, respectively. Concerning combination regimens featuring platinum agents, 61 (41.2%), 58 (39.1%), and 29 patients (19.6%) received pemetrexed, taxanes, and other agents, respectively. Thirty-one patients (20.9%) received bevacizumab in addition to platinum-based chemotherapy. The overall ORR was 37.1% (95% confidence interval [CI] 29.8%–45.2%), and median PFS and OS were 5.6 (95% CI 5.1–6.2 months) and 17.0 months (95% CI 14.6–22.9 months), respectively. The numbers of events for PFS and OS were 115 and 94, respectively. The median observation time was 13.3 months (95% CI 17.2–25.3 months).

**Table 1:** Patient characteristics

	All, n = 148	High GNRI, n = 110	Low GNRI, n = 38	*p*-value
Age, years	65 (36–84)	65 (36–84)	67 (40–79)	0.175
Sex, men	109 (73.6)	30 (27.3)	9 (23.7)	0.831
Smoking status, ever-smoker	111 (75.0)	80 (72.7)	31 (81.6)	0.385
ECOG-PS, 0/1/ ≥ 2	90 (60.8)/42 (28.3)/16 (10.8)	77 (70.0)/29 (23.4)/4 (3.6)	13 (34.2)/13 (34.2)/12 (31.6)	< 0.001
Body mass index, kg/m^2^	21.9 (13.7–30.5)	22.7 (13.7–30.5)	18.5 (14.2–25.0)	< 0.001
Serum albumin (g/dL)	3.7 (2.1–4.9)	3.9 (2.5–4.9)	2.9 (2.1–3.8)	< 0.001
GNRI	100.6 (56.6–124.6)	105.0 (92.5–124.6)	82.0 (55.6–90.5)	< 0.001
Stage, IIIb/IV/recurrence	20 (13.5)/127 (85.8)/1 (0.7)	15 (13.6)/94 (85.5)/1 (0.9)	5 (13.2)/33 (86.8)/0 (0)	1.000
Pathology, squamous/non-squamous	39 (26.4)/109 (73.6)	26 (23.6)/84 (76.4)	13 (34.2)/25 (65.8)	0.208
*EGFR* mutation, positive/wild-type/unknown	16 (10.8)/101 (68.2)/31 (20.9)	15 (13.6)/71 (64.6)/24 (21.8)	1 (2.6)/30 (79.0)/7 (18.4)	0.120
*ALK* fusion gene, positive/wild-type/unknown	1 (0.7)/73 (49.3)/74 (50.0)	1 (0.9)/58 (52.7)/51 (46.4)	0 (0)/15 (39.5)/23 (60.5)	0.396
Comorbidities				
Diabetes	24 (16.2)	20 (18.2)	4 (10.5)	0.319
Chronic pulmonary diseases	22 (14.9)	14 (12.7)	8 (21.1)	0.289
Myocardial infarction	6 (4.1)	5 (4.6)	1 (2.6)	1.000
Peripheral vascular diseases	3 (2.0)	1 (0.9)	2 (5.3)	0.162
Collagen diseases	4 (2.7)	2 (1.8)	2 (5.3)	0.272
Solid tumors	5 (3.4)	3 (2.7)	2 (5.3)	0.603
Liver diseases	4 (2.7)	1 (0.9)	3 (7.9)	0.052
Charlson comorbidity index	0 (0–3)	0 (0–3)	0 (0–3)	0.300
Chemotherapy regimens				0.273
Carboplatin/(nab-)paclitaxel ± bevacizumab	54 (36.5)	41 (37.3)	13 (34.2)	
Carboplatin/pemetrexed ± bevacizumab	45 (30.4)	34 (30.9)	11 (29.0)	
Cisplatin/pemetrexed ± bevacizumab	16 (10.8)	14 (12.7)	2 (5.3)	
Other cisplatin-based therapy	20 (13.5)	10 (9.1)	3 (7.9)	
Other carboplatin-based therapy	13 (8.8)	11 (10.0)	9 (23.7)	
Treatment year, 2000–2010/2011–2020	61 (41.2)/87 (58.8)	44 (40.0)/66 (60.0)	17 (44.7)/21 (55.3)	0.703

Data are expressed as the median (range) or number (%). The *p* values indicate comparisons between high and low GNRI group

*ALK* anaplastic lymphoma kinase, *ECOG-PS* Eastern Cooperative Oncology Group performance status, *EGFR* epidermal growth factor receptor, *GNRI* geriatric nutritional risk index

### Differences in treatments according to the time of initiation of chemotherapy

To evaluate the influence of advances in cancer therapy during the observation period, the patients were divided into two groups according to the date of administration of platinum-based chemotherapy: first decade (from January 2000 to December 2010, n = 61) and second decade (from January 2011 to December 2020, n = 87). The patients treated in the second decade were significantly older (median, 66 years) than those treated in the first decade (median, 64 years; *p* = 0.038). Patients in the second decade had a significantly higher rate of pemetrexed-based regimen treated (62%) and lower rates of treatment with taxane-based (25%) and other regimens (13%) than those treated in the first decade (11%, 59%, and 30%, respectively; *p* < 0.001). Thirty-one (36%) patients treated in the second decade received bevacizumab in addition to platinum-based chemotherapy, whereas no patient received these treatments in the first decade (*p* < 0.001). The patients treated in the second decade exhibited significantly longer PFS (median, 6.0 months) than those treated in the first decade (median, 4.9 months; *p* = 0.004). There was no significant difference in ORR and OS between the first (ORR = 42.5%, median OS = 19.3 months) and second decades (ORR = 29.5%, *p* = 0.122; and median OS = 16.8 months, *p* = 0.521).

### Associations of the GNRI with patient demographics

There was a significant stepwise decrease in the GNRI according to the deterioration of ECOG-PS. Specifically, the median GNRIs (range) in the ECOG-PS 0, 1, and ≥ 2 groups were 104.6 (65.8–124.6), 98.3 (56.6–121.7), and 82.0 (69.4–112.9), respectively (*p* < 0.001). The GNRI was not associated with sex, age, smoking status, tumor histology, clinical stage, or the time of the start of platinum-based chemotherapy. The patients with a high or low GNRI had comparable demographics including comorbidities excluding the significantly better ECOG-PS, higher BMI, and serum albumin level in patients with a high GNRI (all *p* < 0.001; Table 1).

### Association of the GNRI with the efficacy of platinum-based chemotherapy

**Fig. 2:**
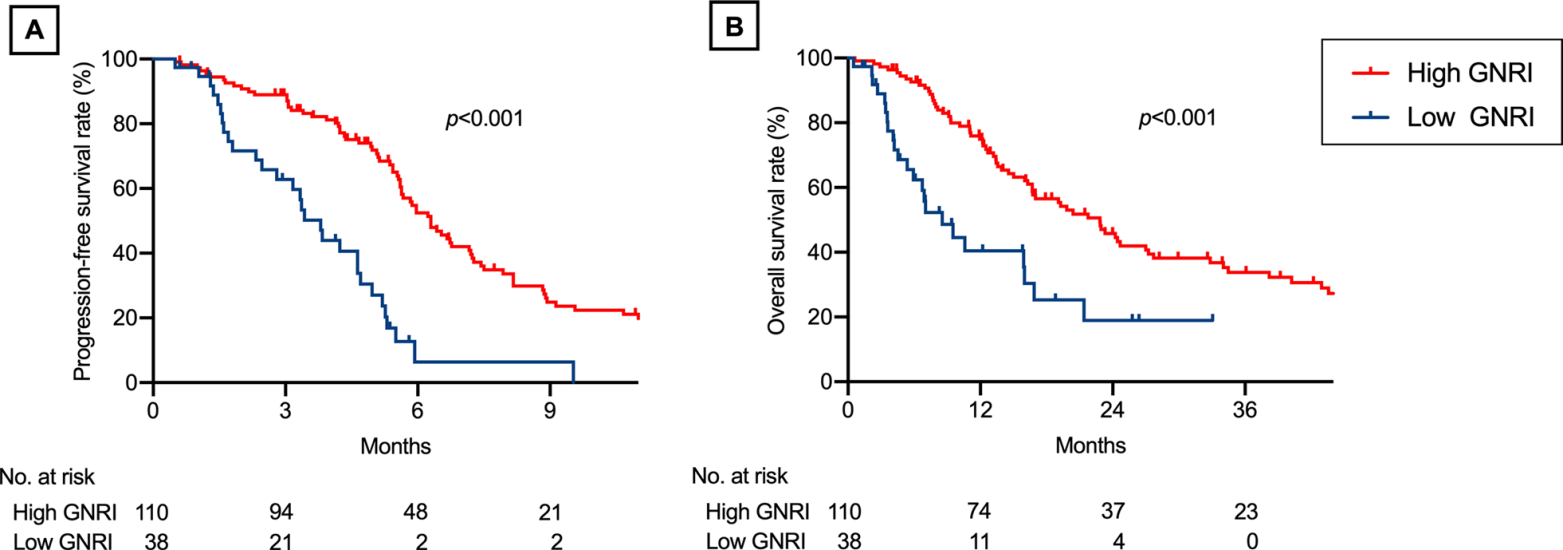
Progression-free and overall survival after first-line platinum-based chemotherapy according to the Geriatric Nutritional Risk Index (GNRI). Kaplan–Meier curves of **a** progression-free survival and **b** overall survival according to the GNRI. Blue and red lines indicate low and high GNRI, respectively

Patients with a high GNRI had significantly longer median PFS (6.3 months, 95% CI = 5.6–7.2 months) than those with a low GNRI (3.8 months; 95% CI = 2.5–4.7 months, *p* < 0.001; Fig. 2a). In univariate Cox proportional hazard analyses, an increased GNRI was predictive of longer PFS, similarly as age < 65 years, good ECOG-PS, receipt of cisplatin, receipt of pemetrexed, and the time of the start of platinum-based chemotherapy (Table 2). In multivariate Cox proportional hazard analyses, only an increased GNRI was an independent predictive factor for longer PFS (Table 2).

**Table 2:** Cox proportional hazard analyses of progression-free survival

	Univariate		Multivariate	
Variables	Hazard ratio (95% CI)	*p*-value	Hazard ratio (95% CI)	*p*-value
Age, ≥ 65 years	1.47 (1.01–2.14)	0.041	1.46 (0.99–2.15)	0.056
Sex, men	1.02 (0.68–1.57)	0.941		
Smoking, ever-smoker	1.27 (0.84–1.96)	0.257		
ECOG-PS*				
0 vs. 1	0.71 (0.47–1.09)	0.114	0.98 (0.63–1.55)	0.928
0 vs. ≥ 2	0.34 (0.18–0.71)	0.006	0.69 (0.34–1.53)	0.340
1 vs. ≥ 2	0.48 (0.24–1.03)	0.059	0.70 (0.35–1.54)	0.358
Charlson comorbidity index*				
0 vs. 1	1.28 (0.83–2.05)	0.271		
0 vs. ≥ 2	0.79 (0.47–1.14)	0.407		
1 vs. ≥ 2	0.62 (0.33–1.18)	0.139		
GNRI, ≥ 92	0.28 (0.18–0.46)	< 0.001	0.35 (0.21–0.58)	< 0.001
Pathology, squamous cell (vs. non-squamous)	1.20 (0.79–1.79)	0.390		
Stage, IIIb (vs. IV/ recurrent)	0.85 (0.44–1.47)	0.575		
Platinum agents, cisplatin (vs. carboplatin)	0.59 (0.35–0.95)	0.029	0.64 (0.37–1.06)	0.086
Non-platinum agents*				
Pemetrexed vs. taxane	0.62 (0.41–0.95)	0.028	0.85 (0.54–1.37)	0.509
Pemetrexed vs. others	0.58 (0.35–0.98)	0.042	0.79 (0.45–1.40)	0.408
Taxane vs. others	0.93 (0.57–1.55)	0.781	0.92 (0.55–1.56)	0.749
Treatment year, 2011–2020 (vs. 2000–2010)	0.56 (0.39–0.84)	0.005	0.65 (0.42–1.01)	0.053

*CI* confidence interval, *ECOG-PS* Eastern Cooperative Oncology Group performance status, *GNRI* Geriatric Nutritional Risk Index, *PD-L1* programmed cell death-ligand 1, *TPS* tumor proportion score

*Hazard ratio was calculated for each pair of three categorical units

Likewise, patients with a high GNRI had significantly longer median OS (22.8 months, 95% CI = 16.7–27.2 months) than those with a low GNRI (8.5 months, 95% CI = 5.4–16.0 months, *p* < 0.001; Fig. 2b). In univariate Cox proportional hazard analyses, an increased GNRI was predictive of longer OS, similarly as age < 65 years, no smoking history, and good ECOG-PS (Table 3). In multivariate Cox proportional hazard analyses, an increased GNRI was predictive of longer OS, similarly as no smoking history and good ECOG-PS (Table 3).

**Table 3:** Cox proportional hazard analyses of overall survival

	Univariate		Multivariate	
Variables	Hazard ratio (95% CI)	*p*-value	Hazard ratio (95% CI)	*p*-value
Age, ≥ 65 years	1.70 (1.11–2.59)	0.014	1.46 (0.94–2.27)	0.093
Sex, men	1.01 (0.65–1.61)	0.959		
Smoking, ever-smoker	1.92 (1.20–3.22)	0.006	1.71 (1.04–2.91)	0.034
ECOG-PS*				
0 vs. 1	0.67 (0.43–1.07)	0.096	0.76 (0.47–1.23)	0.253
0 vs. ≥ 2	0.21 (0.11–0.43)	< 0.001	0.28 (0.14–0.60)	0.002
1 vs. ≥ 2	0.31 (0.16–0.65)	0.003	0.37 (0.18–0.81)	0.014
Charlson comorbidity index*				
0 vs. 1	0.93 (0.58–1.54)	0.762		
0 vs. ≥ 2	1.26 (0.66–2.72)	0.506		
1 vs. ≥ 2	1.36 (0.64–3.13)	0.432		
GNRI, ≥ 92	0.39 (0.24–0.65)	< 0.001	0.51 (0.30–0.89)	0.018
Pathology, squamous cell (vs. non-squamous)	1.18 (0.73–1.84)	0.492		
Stage, IIIb (vs. IV/ recurrent)	1.41 (0.76–2.42)	0.265		
Platinum agents, cisplatin (vs. carboplatin)	0.71 (0.41–1.18)	0.195		
Non-platinum agents*				
Pemetrexed vs. taxane	0.84 (0.54–1.31)	0.437		
Pemetrexed vs. others	1.13 (0.63–2.12)	0.688		
Taxane vs. others	1.35 (0.76–2.53)	0.319		
Treatment year, 2011–2020 (vs. 2000–2010)	1.15 (0.76–1.76)	0.519		

*CI* confidence interval, *ECOG-PS* Eastern Cooperative Oncology Group performance status, *GNRI* Geriatric Nutritional Risk Index

*Hazard ratio was calculated for each pair of three categorical units

Patients with a high GNRI displayed significantly higher ORR (44.5%, 95% CI = 35.6%–53.9%) than those with a low GNRI (15.8%, 95% CI = 7.4%–30.4%, *p* = 0.002). In univariate logistic regression analyses, an increased GNRI, good ECOG-PS, receipt of pemetrexed were predictive of higher ORR. In multivariate logistic regression analyses, an increased GNRI was predictive of the ORR (Table 4).

**Table 4:** Logistic regression analyses of objective response

Variables	Univariate		Multivariate	
	Odds ratio (95% CI)	*p*-value	Odds ratio (95% CI)	*p*-value
Age, ≥ 65 years	0.63 (0.32–1.24)	0.181		
Sex, men	0.80 (0.38–1.71)	0.562		
Smoking, ever-smoker	0.71 (0.33–1.53)	0.380		
ECOG-PS*				
0 vs. 1	1.53 (0.72–3.35)	0.272	1.25 (0.56–2.83)	0.585
0 vs. ≥ 2	5.35 (1.39–35.37)	0.012	2.09 (0.43–15.28)	0.373
1 vs. ≥ 2	3.50 (0.82–24.28)	0.094	1.67 (0.33–12.51)	0.548
Charlson comorbidity index*				
0 vs. 1	1.04 (0.48–2.31)	0.927		
0 vs. ≥ 2	1.64 (0.57–5.47)	0.371		
1 vs. ≥ 2	1.58 (0.48–5.80)	0.457		
GNRI, ≥ 92	4.28 (1.76–12.11)	< 0.001	3.25 (1.22–9.97)	0.018
Pathology, squamous cell (vs. non-squamous)	0.68 (0.30–1.47)	0.331		
Stage, IIIb (vs. IV/ recurrent)	1.15 (0.42–2.98)	0.779		
Platinum agents, cisplatin (vs. carboplatin)	1.49 (0.65–3.39)	0.345		
Non-platinum agents*				
Pemetrexed vs. taxane	1.61 (0.77–3.41)	0.541	1.46 (0.68–3.20)	0.334
Pemetrexed vs. others	2.67 (1.03–7.59)	0.044	2.17 (0.80–6.41)	0.131
Taxane vs. others	1.65 (0.62–4.78)	0.319	1.49 (0.53–4.44)	0.455
Treatment year, 2011–2020 (vs. 2000–2010)	1.77 (0.89–3.59)	0.104		

*CI* confidence interval, *ECOG-PS* Eastern Cooperative Oncology Group performance status, *GNRI* Geriatric Nutritional Risk Index, *N.E.* not estimated

*Odds ratio was calculated for each pair of three categorical units

### Association of the GNRI with the treatment delivery and adverse events of platinum-based chemotherapy

Patients with a high GNRI completed significantly more cycles of platinum-based chemotherapy (median, 4 cycles) and had a higher rate platinum-based chemotherapy completion (75.5%) than those with a low GNRI (median, 2.5 cycles, *p* < 0.001; and 39.5%, *p* < 0.001, respectively; Table 5). Patients with a low GNRI tended to have higher rates of grade ≥ 3 adverse events (55.3%) and dose reduction (39.5%) than those with a high GNRI (38.2%, *p* = 0.087; and 21.8%, *p* = 0.053, respectively).

**Table 5:** Treatment delivery and grade ≥ 3 adverse events

	All, n = 148	High GNRI, n = 110	Low GNRI, n = 38	*p*-value
Cycles of platinum-based therapy	4 (1–8)	4 (1–8)	2.5 (1–6)	< 0.001
Completion of platinum-based chemotherapy	98 (66.2)	83 (75.5)	15 (39.5)	< 0.001
Reasons for discontinuation				0.423
Progressive disease	31 (62.0)	19 (70.4)	12 (52.2)	
Adverse events	5 (14.0)	5 (18.5)	7 (30.4)	
Others	12 (24.0)	3 (11.1)	4 (17.4)	
Treatment delay	21 (14.2)	16 (14.6)	5 (13.2)	1.000
Dose reduction	39 (26.4)	24 (21.8)	15 (39.5)	0.053
Grade ≥ 3 adverse events	63 (42.6)	42 (38.2)	21 (55.3)	0.087

Data are expressed as the median (range) or number (%). The *p* values indicate comparisons between high and low GNRI group

*GNRI* geriatric nutritional risk index

### Association of GNRI with the efficacy of second-line non-platinum chemotherapy

**Fig. 3:**
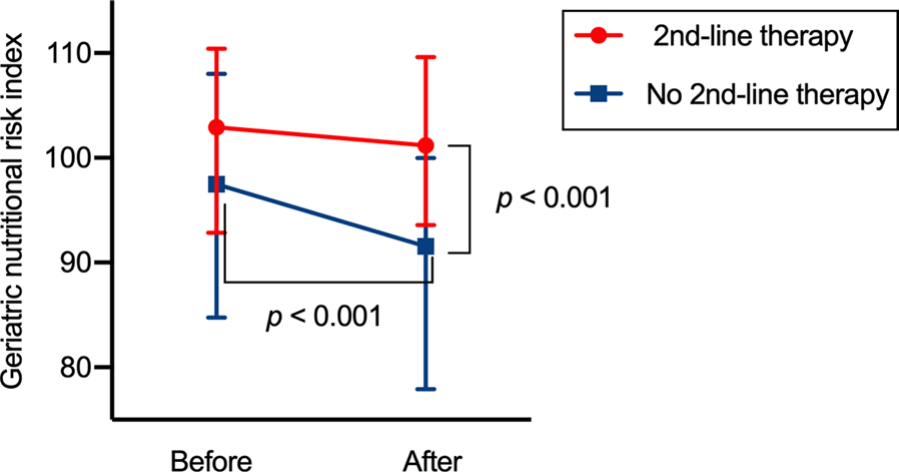
Changes in the Geriatric Nutritional Risk Index between before and after first-line platinum-based chemotherapy. Dots and error bars indicate the median and interquartile range, respectively. Red and blue lines indicate patients who did and did not receive second-line chemotherapy, respectively

**Fig. 4:**
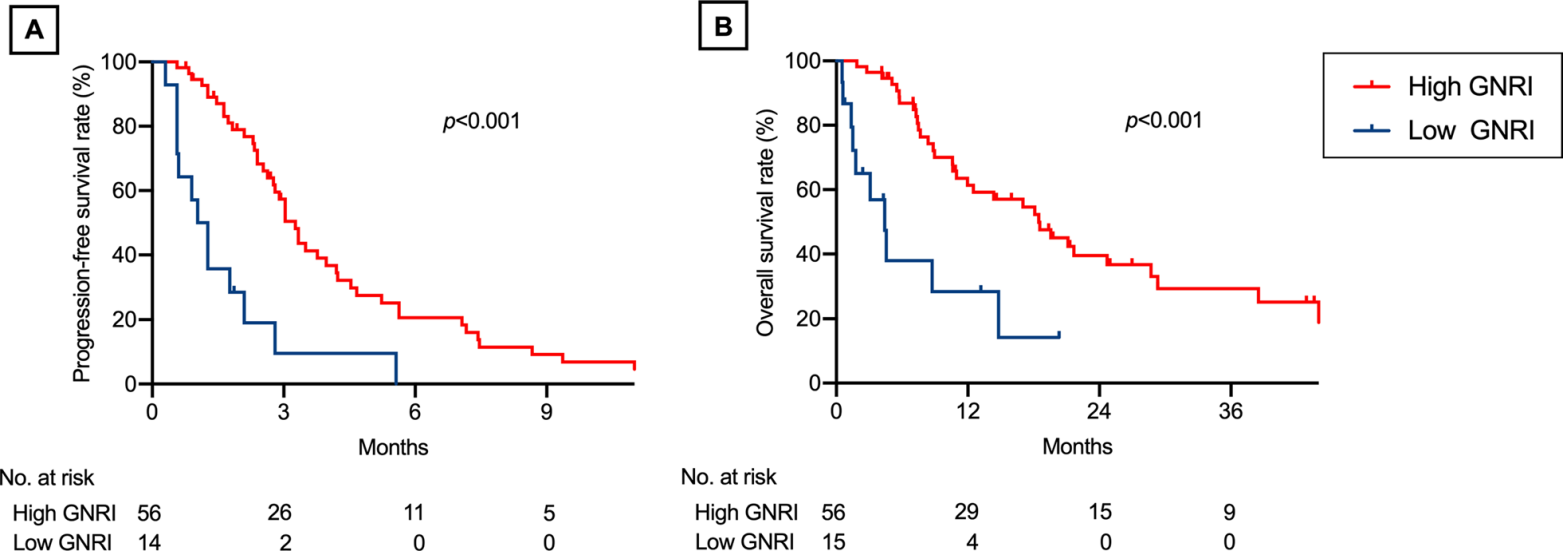
Progression-free and overall survival after second-line non-platinum chemotherapy according to the Geriatric Nutritional Risk Index (GNRI). Kaplan–Meier curves of **a** progression-free survival and **b** overall survival according to the GNRI. Blue and red lines indicate low and high GNRI, respectively

Among the 148 patients who received first-line platinum-based chemotherapy, 71 (48.0%) received second-line non-platinum therapy (2L group). Of those, 47 patients (63.6%) received docetaxel (monotherapy, n = 42; combination with bevacizumab, n = 6; and combination with ramucirumab, n = 1), 12 patients (16.9%) received S-1 (tegafur/gimeracil/oteracil potassium), 4 patients received (5.6%) pemetrexed, and 8 patients (11.3%) received other non-platinum monotherapies. The 2L group had a median GNRI of 101.6 (range 69.7–129.7) at the beginning of the second-line therapy, which was comparable to that at the beginning of first-line therapy (*p* = 0.941; Fig. 3). The patients with a high GNRI at the beginning of second-line therapy exhibited significantly longer median PFS during second-line therapy (3.3 months, 95% CI 2.6–4.2 months) than those with a low GNRI (1.2 months, 95% CI 0.6–2.1 months, *p* < 0.001; Fig. 4a). Likewise, the patients with a high GNRI at the beginning of second-line therapy displayed significantly longer median OS during second-line therapy (18.5 months, 95% CI 11.0–28.7 months) than those with a low GNRI (4.4 months, 95% CI 1.4–14.8 months, *p* < 0.001; Fig. 4b). There was no significant association between the GNRI and ORR during second-line therapy (*p* = 0.324).

On the contrary, among 77 patients who did not receive second-line chemotherapy, 59 evaluable patients had a median GNRI of 91.6 (range 59.0–112.6) at the time of disease progression after first-line chemotherapy, which was significantly lower than that at the beginning the first-line chemotherapy (*p* < 0.001) and significantly lower than that at beginning of the second-line therapy in the 2L group (*p* < 0.001; Fig. 3).

## Discussion

In the current study, we found that an increased pretreatment GNRI was significantly associated with longer PFS and OS following first-line platinum-based chemotherapy in patients with NSCLC independent of ECOG-PS. Furthermore, the GNRI did not change in patients who received second-line chemotherapy until the start of second-line chemotherapy, and the pretreatment GNRI was significantly associated with longer PFS and OS following second-line non-platinum chemotherapy. The GNRI is a simple modality for assessing the nutritional status of patients with cancer. Our data indicated the potential utility of the GNRI for predicting the efficacy of chemotherapy.

Albumin, a component of the GNRI, has several beneficial functions for chemotherapy. After injection into blood, platinum agents bind to albumin and form platinum–albumin complexes. Albumin delivers platinum agents efficiently to tumor tissue via these complexes. Albumin also protects against platinum-associated toxicities by reducing the levels of albumin-free platinum agents that induce cytotoxicity [12–14]. In addition, albumin is known to have immunomodulatory functions in tumor microenvironments. Albumin inhibits tumor progression by reducing excessive inflammatory responses by tumor-associated neutrophils [26–29]. Furthermore, albumin reduces oxidative stress in tumor microenvironments via its anti-oxidant properties [26, 27]. Oxidative stress induces immunosuppression in tumor microenvironments by altering cytokine signaling, increasing immunosuppressive immune cell activity, and attenuating cytotoxic lymphocytes, resulting in tumor-favorable immunity [31, 32]. It is reported that the Prognostic Nutritional Index, which is calculated using serum albumin and the peripheral blood lymphocyte count, is positively correlated with tumor-infiltrating lymphocyte counts in surgically resected esophageal cancer and squamous cell lung cancer specimens [33, 34].

Body weight is an important component of cancer cachexia. In addition to the reduction of adipose tissue, the loss of muscle mass is also a cause of weight loss in patients with cancer, resulting in functional impairments and increased mortality [6–9]. In addition, reduced food intake, which both a cause and consequence of cancer cachexia, leads to the deprivation of essential nutrients, some of which are reported to potentially enhance anti-tumor immunity [5, 35–37]. Furthermore, systemic inflammation and metabolic changes, the underlying mechanisms of cancer cachexia, attenuate anti-tumor immunity and promote tumor progression [38, 39]. Body weight loss attributable to cancer cachexia reflects the attenuation of anti-tumor immunity and decreases the therapeutic efficacy of chemotherapy.

Given the multiple roles of the nutritional status in the general health condition, transportation of anti-cancer drugs, protection of tissue from chemotherapy, and cancer immunity, a high GNRI has the potential to increase the efficacy of chemotherapy, decrease toxicities, and therefore prolong survival. In addition to the higher ORR, patients with a high GNRI had less severe adverse events and less frequently required dose reduction (although not significant), which might explain the higher number of chemotherapy cycles and the longer PFS and OS.

The current study had three main limitations. First, this was a retrospective study with a limited number of patients. It is possible that some potential biases and/or alpha errors affected the results of the current study. Second, the optimal evaluation for nutritional status in patients with cancer is unknown. The GNRI was used in the current study because it can be calculated using two simple values that are readily available in clinical practice. However, several other nutritional indexes using various combinations of factors in addition to (or instead of) albumin and body weight, such as prealbumin, cholesterol, neutrophil, lymphocyte, C-reactive protein, or body mass index, are also available [4]. Additionally, the cutoff of 92 in GNRI was provisional, and it should be validated in the further studies. Third, the current study evaluated cytotoxic chemotherapy. ICIs are increasingly used as new standard treatments for cancers, including NSCLC [40, 41]. Furthermore, several regimens combining chemotherapy with one or more ICIs have been developed [42, 43]. It is reported that GNRI is associated with the efficacy of ICI monotherapy [25]. Thus, GNRI is expected to be predictive of the efficacy of novel combinations of chemotherapy and ICIs. Further studies are needed to elucidate the predictive utility of the nutritional status and the optimal nutritional index for novel cancer therapies.

## Conclusions

In conclusion, increased GNRI was associated with better PFS and OS following first-line platinum-based chemotherapy and second-line non-platinum therapy in patients with NSCLS independent of ECOG-PS. Assessments of nutritional status may be useful for predicting the efficacy of chemotherapy.

## Supplementary Information

**Additional file 1**.** Supplementary Figure**. Progression-free and overall survival after platinum-based chemotherapy according to four levels of the geriatric nutritional risk index (GNRI).

## Data Availability

The datasets used and/or analyzed during the current study are available from the corresponding author on reasonable request.

## Abbreviations

ALK: Anaplastic lymphoma kinase
CI: Confidence interval
ECOG-PS: Eastern Cooperative Oncology Group performance status
EGFR: Epidermal growth factor receptor
GNRI: Geriatric Nutritional Risk Index
ICIs: Immune checkpoint inhibitors
NSCLC: Non-small-cell lung cancer
ORR: Overall response rate
OS: Overall survival
PFS: Progression-free survival

## References

[1] Galmés S, Serra F, Palou A (2020). Current state of evidence: Influence of nutritional and nutrigenetic factors on immunity in the COVID-19 pandemic framework. Nutrients.

[2] Faverio P, De Giacomi F, Bodini BD, Stainer A, Fumagalli A, Bini F (2021). Nontuberculous mycobacterial pulmonary disease: an integrated approach beyond antibiotics. ERJ Open Res.

[3] Healy C, Munoz-Wolf N, Strydom J, Faherty L, Williams NC, Kenny S (2021). Nutritional immunity: the impact of metals on lung immune cells and the airway microbiome during chronic respiratory disease. Respir Res.

[4] Baldessari C, Guaitoli G, Valoriani F, Bonacini R, Marcheselli R, Reverberi L (2021). Impact of body composition, nutritional and inflammatory status on outcome of non-small cell lung cancer patients treated with immunotherapy. Clin Nutr ESPEN.

[5] Ramalho R, Rao M, Zhang C, Agrati C, Ippolito G, Wang FS (2020). Immunometabolism: new insights and lessons from antigen-directed cellular immune responses. Semin Immunopathol.

[6] Fearon KC, Voss AC, Hustead DS (2006). Definition of cancer cachexia: Effect of weight loss, reduced food intake, and systemic inflammation on functional status and prognosis. Am J Clin Nutr.

[7] Vanhoutte G, Van De Wiel M, Wouters K, Sels M, Bartolomeeussen L, De Keersmaecker S (2016). Cachexia in cancer: what is in the definition?. BMJ Open Gastroenterol.

[8] Prado CMM, Baracos VE, McCargar LJ, Reiman T, Mourtzakis M, Tonkin K (2009). Sarcopenia as a determinant of chemotherapy toxicity and time to tumor progression in metastatic breast cancer patients receiving capecitabine treatment. Clin Cancer Res.

[9] Ross PJ, Ashley S, Norton A, Priest K, Waters JS, Eisen T (2004). Do patients with weight loss have a worse outcome when undergoing chemotherapy for lung cancers?. Br J Cancer.

[10] Temel JS, Abernethy AP, Currow DC, Friend J, Duus EM, Yan Y (2016). Anamorelin in patients with non-small-cell lung cancer and cachexia (ROMANA 1 and ROMANA 2): results from two randomised, double-blind, phase 3 trials. Lancet Oncol.

[11] Wang Z, Aguilar EG, Luna JI, Dunai C, Khuat LT, Le CT (2019). Paradoxical effects of obesity on T cell function during tumor progression and PD-1 checkpoint blockade. Nat Med.

[12] Sooriyaarachchi M, Narendran A, Gailer J (2011). Comparative hydrolysis and plasma protein binding of cis-platin and carboplatin in human plasma in vitro. Metallomics.

[13] Park CR, Kim HY, Song MG, Lee Y (2020). Efficacy and safety of human serum albumin—cisplatin complex in U87MG xenograft mouse models. Int J Mol Sci.

[14] Liang Y, Xu L, Yang H, Xu W, Hu R, Fan X (2021). Analysis on the interaction and binding properties of daphnoretin and human serum albumin in the presence of cisplatin: multi-spectroscopic methods and docking simulation. Eur J Pharm Sci.

[15] Bouillanne O, Morineau G, Dupant C, Coulombel I, Vincent JP, Nicolis I (2005). Geriatric Nutritional Risk Index: a new index for evaluating at-risk elderly medical patients. Am J Clin Nutr.

[16] Matsukuma Y, Tanaka S, Taniguchi M, Nakano T, Masutani K, Hirakata H (2019). Association of geriatric nutritional risk index with infection-related mortality in patients undergoing hemodialysis: the Q-Cohort Study. Clin Nutr.

[17] Wei L, Xie H, Li J, Li R, Chen W, Huang L (2020). The prognostic value of geriatric nutritional risk index in elderly patients with severe community-acquired pneumonia: a retrospective study. Medicine (Baltimore).

[18] Dong CH, Chen SY, Zeng HL, Yang B, Pan J (2021). Geriatric nutritional risk index predicts all-cause mortality in patients with heart failure: a systematic review and meta-analysis. Clinics.

[19] Matsumura T, Mitani Y, Oki Y, Fujimoto Y, Ohira M, Kaneko H (2015). Comparison of Geriatric Nutritional Risk Index scores on physical performance among elderly patients with chronic obstructive pulmonary disease. Hear Lung J Acute Crit Care.

[20] Kanno H, Goto Y, Sasaki S, Fukutomi S, Hisaka T, Fujita F (2021). Geriatric nutritional risk index predicts prognosis in hepatocellular carcinoma after hepatectomy: a propensity score matching analysis. Sci Rep.

[21] Tang Q-N, Qiu H-Z, Sun X-Q, Guo S-S, Liu L-T, Wen Y-F (2021). Geriatric nutritional risk index as an independent prognostic factor in locally advanced nasopharyngeal carcinoma treated using radical concurrent chemoradiotherapy: a retrospective cohort study. Ann Transl Med.

[22] Chang LW, Hung SC, Li JR, Chiu KY, Yang CK, Chen CS (2021). Geriatric Nutritional Risk Index as a prognostic marker for patients with metastatic castration-resistant prostate cancer receiving docetaxel. Front Pharmacol.

[23] Shoji F, Matsubara T, Kozuma Y, Haratake N, Akamine T, Takamori S (2017). Preoperative Geriatric Nutritional Risk Index: a predictive and prognostic factor in patients with pathological stage I non-small cell lung cancer. Surg Oncol.

[24] Peng SM, Yu N, Ren JJ, Xu JY, Chen GC, Yang JR, et al. The geriatric nutritional risk index as a prognostic factor in patients with advanced non-small-cell lung cancer. Nutr Cancer. 2020; :33356605. 10.1080/01635581.2020.1865423 33356605

[25] Sonehara K, Tateishi K, Araki T, Komatsu M, Yamamoto H, Hanaoka M (2021). Prognostic value of the geriatric nutritional risk index among patients with previously treated advanced non-small cell lung cancer who subsequently underwent immunotherapy. Thorac Cancer.

[26] Wiedermann CJ (2021). Hypoalbuminemia as surrogate and culprit of infections. Int J Mol Sci.

[27] Ferrer R, Mateu X, Maseda E, Yébenes JC, Aldecoa C, De Haro C (2018). Non-oncotic properties of albumin. A multidisciplinary vision about the implications for critically ill patients. Expert Rev Clin Pharmacol.

[28] Erpenbeck L, Schön MP (2017). Neutrophil extracellular traps: Protagonists of cancer progression?. Oncogene.

[29] Neubert E, Senger-Sander SN, Manzke VS, Busse J, Polo E, Scheidmann SEF (2019). Serum and serum albumin inhibit in vitro formation of Neutrophil Extracellular Traps (NETs). Front Immunol.

[30] Charlson ME, Pompei P, Ales KL, MacKenzie CR (1987). A new method of classifying prognostic comorbidity in longitudinal studies: development and validation. J Chronic Dis.

[31] Augustin RC, Delgoffe GM, Najjar YG (2020). Characteristics of the tumor microenvironment that influence immune cell functions: Hypoxia, oxidative stress, metabolic alterations. Cancers (Basel).

[32] Maj T, Wang W, Crespo J, Zhang H, Wang W, Zhao L (2018). Oxidative stress controls regulatory T cell apoptosis and suppressor activity and PD-L1-blockade resistance in tumor. Nat Immunol.

[33] Okadome K, Baba Y, Yagi T, Kiyozumi Y, Ishimoto T, Iwatsuki M (2020). Prognostic Nutritional Index, tumor-infiltrating lymphocytes, and prognosis in patients with esophageal cancer. Ann Surg.

[34] Kitahara H, Shoji F, Akamine T, Kinoshita F, Haratake N, Takenaka T (2021). Preoperative prognostic nutritional index level is associated with tumour-infiltrating lymphocyte status in patients with surgically resected lung squamous cell carcinoma. Eur J Cardio-Thoracic Surg.

[35] Kishton RJ, Sukumar M, Restifo NP (2017). Metabolic regulation of T cell longevity and function in tumor immunotherapy. Cell Metab.

[36] Mok EHK, Lee TKW (2020). The pivotal role of the dysregulation of cholesterol homeostasis in cancer: implications for therapeutic targets. Cancers (Basel).

[37] Dyck L, Lynch L (2018). Cancer, obesity and immunometabolism—connecting the dots. Cancer Lett.

[38] Tall AR, Yvan-Chrvet L (2015). Cholesterol inflammation and innate immunity. Nat Rev Immunol.

[39] Newton R, Priyadharshini B, Turka LA (2016). Immunometabolism of regulatory T cells. Nat Immunol.

[40] Borghaei H, Paz-Ares L, Horn L, Spigel DR, Steins M, Ready NE (2015). Nivolumab versus docetaxel in advanced nonsquamous non-small-cell lung cancer. N Engl J Med.

[41] Brahmer J, Reckamp KL, Baas P, Crinò L, Eberhardt WEE, Poddubskaya E (2015). Nivolumab versus docetaxel in advanced squamous-cell non-small-cell lung cancer. N Engl J Med.

[42] Gandhi L, Rodríguez-Abreu D, Gadgeel S, Esteban E, Felip E, De Angelis F (2018). Pembrolizumab plus chemotherapy in metastatic non-small-cell lung cancer. N Engl J Med.

[43] Hellmann MD, Paz-Ares L, Bernabe Caro R, Zurawski B, Kim S-W, Carcereny Costa E (2019). Nivolumab plus Ipilimumab in advanced non-small-cell lung cancer. N Engl J Med.

