# Supplementary material for: Association of the Geriatric Nutritional Risk Index with the survival of patients with non-small-cell lung cancer after platinum-based chemotherapy
Source: BMC Pulm Med. 2021 Dec 11;21:409. doi: 10.1186/s12890-021-01782-2 (PMC8665565; doi:10.1186/s12890-021-01782-2)
Supplement: Supplementary file 1 — Additional file 1. Supplementary Figure. Progression-free and overall survival after platinum-based chemotherapy according to four levels of the geriatric nutritional risk index (GNRI). [file 12890_2021_1782_MOESM1_ESM.pdf]

Supplementary Figures

Association of geriatric nutritional risk index with the survival of patients with non-small-cell lung cancer after platinum-based chemotherapy

Authors: Masato Karayama, Yusuke Inoue, Hideki Yasui, Hironao Hozumi, Yuzo Suzuki, Kazuki Furuhashi, Tomoyuki Fujisawa, Noriyuki Enomoto, Yutaro Nakamura, Naoki Inui, Takafumi Suda

Supplementary Figure. Progression-free and overall survival after platinum-based chemotherapy according to the geriatric nutritional risk index (GNRI)

Kaplan–Meier curves of A) progression-free survival and B) overall survival according to four levels of GNRI. Blue, green, orange, and red lines indicate GNRI <82, ≥82 to <92, ≥92 to <98, and ≥98, respectively.

A

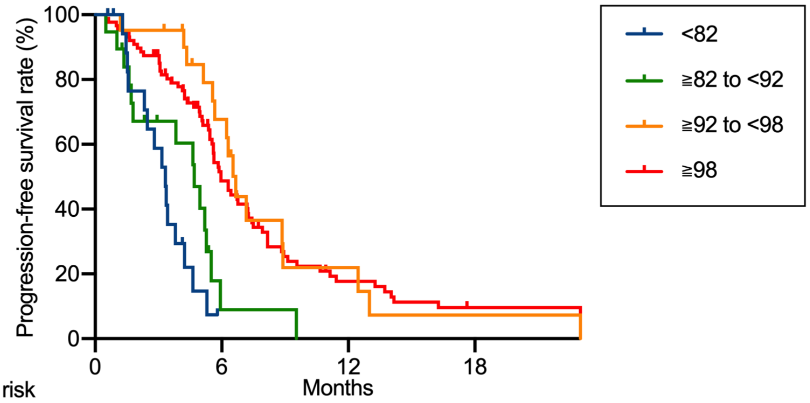

No. at risk

GNRI	0	6	12	18
≧98	88	36	12	6
≧92 to <98	22	13	4	2
≧82 to <92	19	2	0	0
<82	19	0	0	0

B

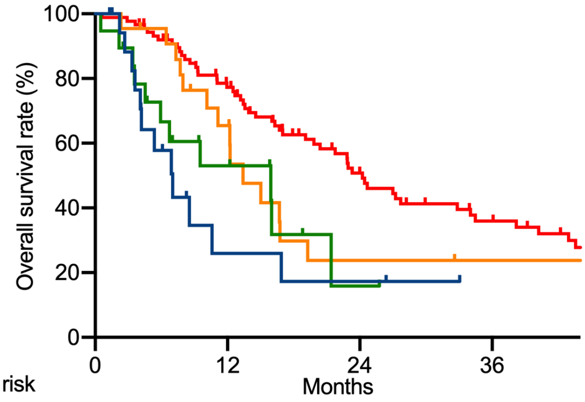

No. at risk

GNRI	0	12	24	36
≧98	88	62	36	21
≧92 to <98	22	13	5	4
≧82 to <92	19	8	2	0
<82	19	4	3	0
